# 2462. The impact of the COVID-19 pandemic on hospital-acquired infections and multidrug-resistant organisms, and comparison with seasonal influenza

**DOI:** 10.1093/ofid/ofad500.2080

**Published:** 2023-11-27

**Authors:** Halima Dabaja-Younis, Zmira Silman, Jalal Tarabeia, Khetam Hussein

**Affiliations:** Rambam Health Care Campus, Haifa, Hefa, Israel; Rambam Health Care Campus, Haifa, Hefa, Israel; Rambam Health Care Campus, Haifa, Hefa, Israel; Rambam Health care campus, Haifa, HaZafon, Israel

## Abstract

**Background:**

Effective preventive measures can curb the spread of hospital-acquired infections (HAIs) and multidrug-resistant organisms (MDROs). However, the impact of coronavirus disease 2019 (COVID-19) and measures taken during it on these infections remains unclear. This study aimed to evaluate the impact of COVID-19 pandemic on HAIs and MDROs and compare it to seasonal influenza.

**Methods:**

A retrospective cohort study that used prospectively collected data from a tertiary referral hospital in Haifa, Israel. The study spanned from 2016 to 2021 and compared the period before and after COVID-19 and the influenza season (December-February) and the non-influenza season (March-November) in terms of the rate of hospital-acquired bloodstream infections (HA-BSI), MDROs, and *Clostridium difficile* infections (CDI) per 10,000 hospital days (HD). The study also measured the rate of central line-associated BSI (CLABSI) per 1,000 catheter days (CD) and the hand hygiene compliance (HHC) rates.

**Results:**

During the COVID-19 period, HAIs and MDROs decreased compared to before: methicillin-resistant *Staphylococcus aureus* (4.2 vs. 6.9/10,000 HD; p< 0.001), carbapenem-resistant *Acinetobacter baumani* (2.2 vs. 3.1/10,000 HD; p=0.02), and nosocomial CDI (3 vs. 4.6/10,000 HD; p< 0.001). However, there were higher rates of carbapenem-resistant *Enterobacteriaceae* (4.6 vs. 2.7/10,000 HD; p< 0.001) and HA-BSI (29.7 vs. 27.3/10,000 HD; p=0.006) in the COVID-19 period. CLABSI rates were not significantly different (2.3 vs. 2.7/1000 CD; p=0.910). HHC rates were 70% in both periods (p=0.151), and there were no significant differences in rates of MDROs, CDI, HA-BSI, or CLABSI (p=0.233, 0.675, 0.267, and 0.563, respectively) between influenza and non-influenza seasons.

The median acquisition rate of multi-drug resistant organisms and Clostridium difficile infections before and during the COVID-19 pandemic and during or between influenza seasons

**Figure:**
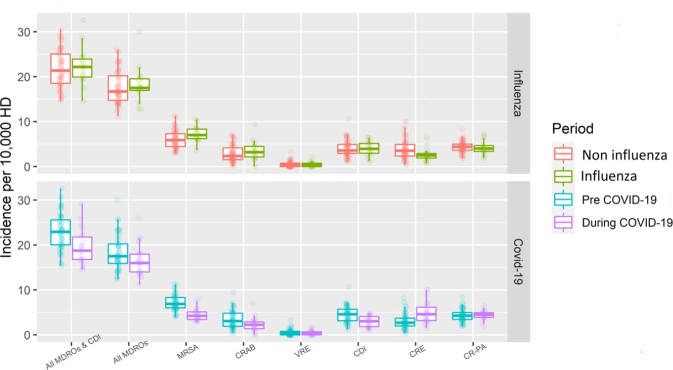

**Conclusion:**

During the COVID-19 period, there was a significant decrease in rates of HAIs and MDROs, accompanied by an increase in CRE rates, which could be attributed to the nationwide increase in CRE rates in Israel since 2016. The decrease in these infections was not attributable to improvement in HHC, suggesting that other factors may be involved. Furthermore, influenza did not show an impact on these infections, likely due to the particular way it is perceived by healthcare workers.

**Disclosures:**

**All Authors**: No reported disclosures

